# Peptide libraries for the comprehensive coverage of the tumor mutanome for immune monitoring and immuno therapy

**DOI:** 10.1186/2051-1426-3-S2-P265

**Published:** 2015-11-04

**Authors:** Paul von Hoegen, Tobias Knaute, Holger Wenschuh, Ulf Reimer

**Affiliations:** 1 JPT Peptide Technologies GmbH, Lasne, Belgium; 2 JPT Innovative Peptide Technologies, Berlin, Germany; 3 JPT Peptide Technologies GmbH, Berlin, Germany

## 

Many cancers are associated with functional mutations which now can be readily identified using new DNA sequencing methods. Sequence information is now used for the generation of personalized cancer vaccines with promising clinical benefit [1]. Intelligent ways to select the relevant, tumor-specific protein-coding mutations are applied to filter the large number of identified mutation to a manageable number. Besides, there is an increasing data pool of mutations, both germline and somatic, the latter frequently even with attached additional information on the tissue and histology.

Even though the use of actual sequence of an individual patient is the optimal basis for treatment and immune monitoring it is quite expensive relatively slow to obtain this information. As a fast tool with broad applicability across patient cohorts peptide libraries could find an application in cases where the individual sequence is not (yet) available.

Pools of antigen peptides are frequently for T cell stimulation in T cell assays and even for immune stimulation for treatment. Such peptide libraries are well suited for presenting sequence diversity. However, libraries used today normally consist of overlapping peptides of a wild-type antigen sequence. The concept was already extended for the presentation of sequence diversity in antigens of pathogens as HIV where sequence diversity between individual viruses poses many challenges. For the ENV protein an increase in sequence coverage from 10 % to 34 % was achieved. Due to the limited number of peptides a complex filtering of sequence data has to be applied to enrich the library with peptides carrying different tumor-specific and ideally functionally relevant mutations.

Our aim is to generate peptide libraries for specific antigens which represent a generic cancer mutanome applicable to a large number of individual patients based on available databases as the catalogue of somatic mutations in cancer and epitope information from the immune epitope database and prediction algorithms.

One library type can contain the full length sequence of the antigen enriched by peptides representing overlapping scans for relevant mutations. Alternatively, known or predicted epitopes can be selected and the mutated epitopes can be included. However, mutations can lead to the formation of new epitopes and mutated epitopes can escape from MHC-binding. Epitope prediction can help to account for these effects.

We have built a pipeline for the efficient generation of such libraries which will be manufactured.

